# Periorbital infections and conjunctivitis due to Panton-Valentine Leukocidin (PVL) positive *Staphylococcus aureus* in children

**DOI:** 10.1186/s12879-018-3281-8

**Published:** 2018-08-06

**Authors:** Pia-Alice Hoppe, Leif G. Hanitsch, Rasmus Leistner, Michaela Niebank, Christoph Bührer, Horst von Bernuth, Renate Krüger

**Affiliations:** 1 Department of Pediatric Pneumology, Immunology and Intensive Care, Charité - Universitätsmedizin Berlin, Augustenburger Platz 1, 13353 Berlin, Germany; 2 Department of Medical Immunology, Charité - Universitätsmedizin Berlin, Augustenburger Platz 1, 13353 Berlin, Germany; 3 Department of Hygiene, Charité - Universitätsmedizin Berlin, Germany, Augustenburger Platz 1, 13353 Berlin, Germany; 4 Department of Internal Medicine/Infectious Diseases and Pulmonary Medicine, Charité -Universitätsmedizin Berlin, Augustenburger Platz 1, 13353 Berlin, Germany; 5 Department of Neonatology, Charité - Universitätsmedizin Berlin, Augustenburger Platz 1, 13353 Berlin, Germany; 6 Sozialpädiatrisches Zentrum, Charité - Universitätsmedizin Berlin, Berlin, Germany; 7 Labor Berlin GmbH, Fachbereich Immunology, Charité -Vivantes, Berlin, Germany; 8 Berlin Center for Regenerative Therapies (BCRT), Charité - Universitätsmedizin Berlin, Augustenburger Platz 1, 13353 Berlin, Germany

**Keywords:** Panton-Valentine Leucocidin, PVL, *Staphylococcus aureus*, Hordeolum, Pediatrics, Eye infection, Lid abscess

## Abstract

**Background:**

Colonisation with Panton-Valentine Leukocidin expressing strains of *Staphylococcus aureus* (PVL + SA) is characterised by recurrent skin and soft tissue infections. While periorbital and orbital infections are common in children and frequently caused by *S. aureus* the role of PVL + SA in recurrent eye infections has not been studied.

This study aimed to detect and report frequency and recurrence of periorbital or orbital infections as additional symptoms of PVL + SA colonisation in children.

**Methods:**

We conducted a retrospective cohort study of pediatric patients who were treated for PVL + SA skin and soft tissue infection in our in- and outpatient clinics in Berlin, Germany from January 2012 to January 2017. We identified cases with periorbital or orbital infections in the year prior to the first PVL + SA evidence. In these cases, we conducted follow-up interviews by phone to determine recurrence of symptoms after the completion of decolonisation procedures.

**Results:**

Fifty pediatric patients (age range: one week to 17 years) were evaluated and treated for PVL + SA infections in the reported time period. 19 patients (38%) reported periorbital infection or conjunctivitis, with recurrent hordeola as the most frequent finding (*n* = 9; 18%). Reappearance of hordeola (*n* = 5) was associated with recurrence of skin and soft tissue infections and/or de novo detection of PVL + SA. No further hordeola or other eye infections occurred after successful decolonisation.

**Conclusion:**

Our findings suggest a frequent involvement of periorbital skin in children with PVL + SA infections. Pediatric patients with recurrent periorbital infections might benefit from PVL + SA screening and consecutive decolonisation procedures.

## Background

Periorbital and orbital infections such as hordeola, conjunctivitis, preseptal and orbital cellulitis are common infections of the eye frequently caused by *Staphylococcus aureus (S. aureus)* [1]. These infections are often the result of ascending infections from the nasopharynx, lacrimal sac or sinuses, common sites of *S. aureus* colonisation [2–4]. A hordeolum is an acute infection of the glands of the eyelid. Hordeola are common, however, there is no exact data on the incidence in children or adults. Due to cosmetic reasons, pain, itching and swelling recurrent hordeola can have a negative impact on a child’s well-being. A recent Cochrane analysis demonstrated a lack of standardised therapeutic guidelines for single or recurrent hordeola [5].

*S. aureus* is the predominant cause for skin and soft tissue infections (SSTI) in the world [6]. About 30% of the general population carry the facultative pathogenic commensal on their skin, mucosa and conjunctiva [7–9]. It is primarily associated with non-invasive infections such as skin abscesses, furunculosis and wound infections, less frequently with more severe infections such as sepsis, osteomyelitis and pneumonia [10]. Nasal colonisation with *S. aureus* has been described as an important risk factor in developing SSTI and eye infections [2, 11–13].

Since the 1990s there has been an increase of infections with *S. aureus* strains expressing the exotoxin Panton-Valentine Leukocidin (PVL + SA), which are mainly characterised by recurrent SSTI [14].

Both methicillin-sensitive (MSSA) as well as methicillin-resistant (MRSA) strains of *S. aureus* express the leukocidin encoded by bacteriophage genes lukS and lukF [15]. In the US, most cases reported are caused by community acquired methicillin resistant strains (CA-MRSA). Although a rise of PVL + CA-MRSA has been reported, PVL expression is still mainly associated with MSSA in Europe [16–19].

In our routine clinical care a remarkable number of children treated for PVL + SA SSTI reported multiple, recurrent and bothersome hordeola. To our knowledge, no studies on pediatric patients with PVL + SA skin infection explored the involvement of the periorbital skin or orbita. To date, there are several case reports on severe eye infections with PVL + CA-MRSA, but none with PVL + MSSA [20–22]. Reports on PVL + SA in non-threatening periorbital infections are scarce.

This study aimed to detect and report frequency and recurrence of periorbital or orbital infections as additional symptoms of PVL + SA colonisation in children.

## Methods

We conducted a retrospective cohort study of pediatric patients who were treated for PVL + SA SSTI in our pediatric in- and outpatient clinics from January 2012 to January 2017. At first presentation in our outpatient clinic, parents of affected children underwent standardized interviews comprising questions on localization and frequency of SSTI and the occurrence and frequency of hordeola up to one year prior to first evidence of PVL + SA infections in the affected child. Screening swabs from anterior nares and oropharynx were taken in each patient. Patients who had been tested positive for PVL + SA in at least one sample (naso-pharyngeal screening swabs and/or swabs from other location, e.g. material from SSTI) were included in this study. In patients with periorbital infection at presentation conjunctival swabs were taken. *S. aureus* was detected by standard bacterial culture. Antimicrobial susceptibilities were determined using a Vitek® 2 automated system and the standard criteria of the European Committee on Antimicrobial Susceptibility Testing (EUCAST). The presence of the genes encoding PVL (lukS and lukF) was assessed by polymerase chain reaction (PCR) as described previously [23]. We excluded patients who had been treated for PVL + SA based on clinical data (typical symptoms plus PVL + SA evidence in household members) but had been tested negative for PVL + SA in the screening swabs.

Data were collected from electronic medical records and archived files. Our main research parameter was the occurrence of an eye infection within 12 months prior to the first PVL + SA detection. We defined *eye infection* as self-report or diagnosis of at least one periorbital or orbital infection. We defined *recurrent infection* as more than two episodes during the reported time period. All patients received decolonisation measures (consisting of: topical application of mupirocin nasal ointment and antiseptic treatment of mouth, hair, skin and housing space for at least five days). *Successful decolonisation* was defined as two negative screening swabs plus no recurrence of SSTI. In patients with a history of eye infection we conducted follow up interviews by phone at least two months after completion of decolonisation measures to detect any recurrence of symptoms after two months. Data were processed using Microsoft® Excel® 2013.

## Results

**Fig. 1:**
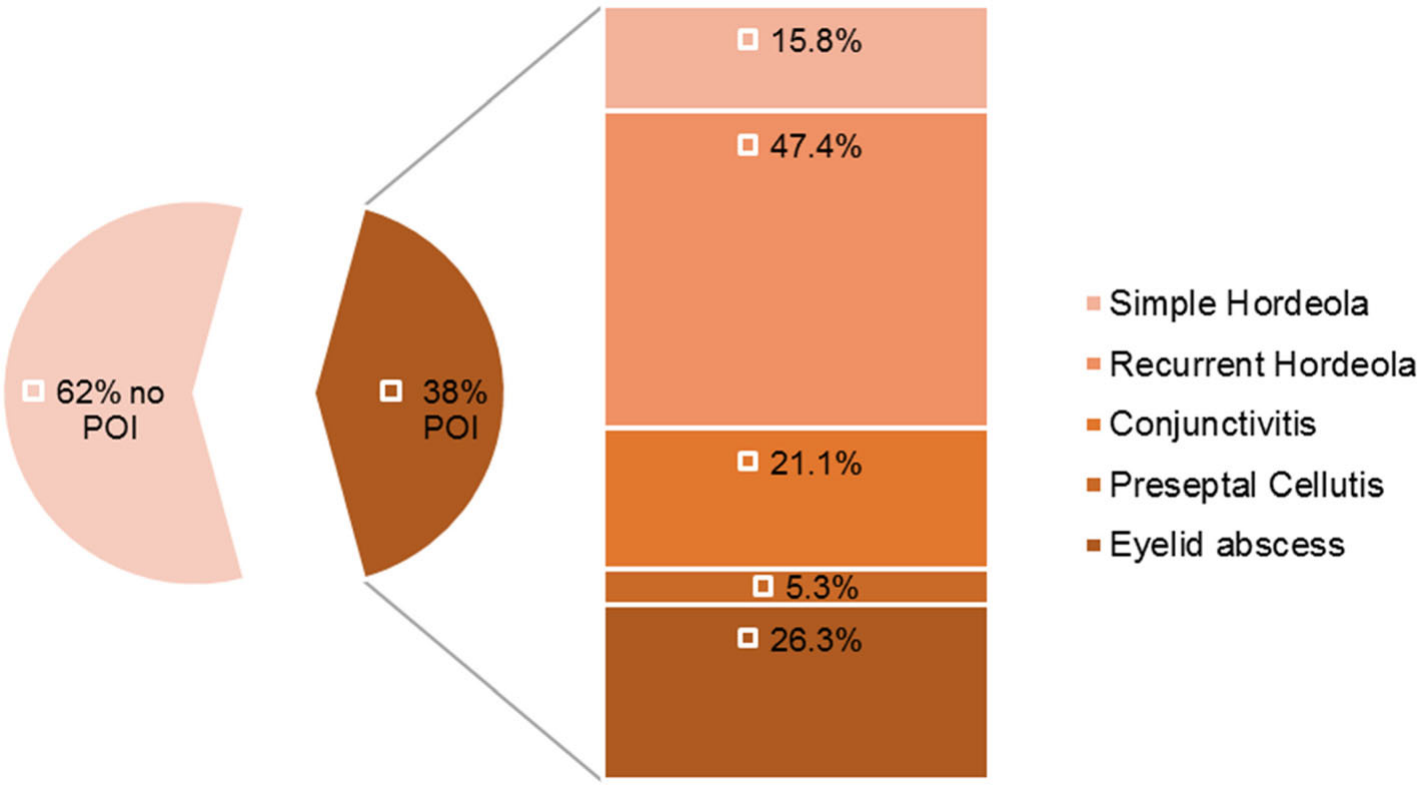
Presentation of eye infection in PVL positive children. Circle: Proportion of PVL+ children (*n* = 50) with or without periorbital infection (POI). Bar: Proportion of types of POI reported by the POI cohort

From January 2012 to January 2017 72 children had received treatment for PVL + SA associated SSTI in our clinic. 22 of these children were treated based on clinical data alone and thus excluded from our study. The remaining 50 patients were included. Median age was 6 years (Range: one week - 17 years). 54% of patients were male (*n* = 27) and 46% female (*n* = 23). Seven children (14%) were infected with PVL + MRSA, all others (86%) with PVL + MSSA. In one patient (age: 17) with multiple hordeola at presentation, PVL + SA was cultured from conjunctival swabs. 19 patients (38%) reported periorbital infections. The characteristics of these patients are summarised in Table 1. Patients suffered from eyelid abscesses (*n* = 5), preseptal cellulitis (*n* = 1), conjunctivitis (*n* = 3) and hordeola (*n* = 12). Nine of these patients (18%) reported recurrent hordeola, with the onset of hordeola with a temporal connection to the onset of SSTI (Fig. 1)*.* Four patients were lost to follow-up. In five patients eye infection reappeared, however, this was associated with recurrence of SSTI in all five cases, with de novo detection of PVL + SA in two cases. In the remaining ten patients no further hordeola or periorbital infections occurred after successful decolonisation (follow-up: 10 weeks to 36 months).

**Table 1:** Characteristics of PVL positive children with eye infections

Patient	Age at diagnosis	MRSA	Hordeola	Conjunctivitis	Preseptal cellulitis	Eyelid abscess	Recurrence of SSTI	Recurrence of eye infection	De novo detection
No.	months (years)	yes/no	n	n	n	n	yes/no	yes/no	yes/no/not tested
1	114 (9)	no	> 2	0	0	0	no	no	not tested
2	1 (0)	no	0	2	1	0	no	no	not tested
3	155 (12)	no	> 2	0	0	0	no	no	not tested
4	117 (9)	no	> 2	0	0	0	yes	yes	not tested
5	16 (1)	no	1	0	0	0	yes	yes	yes
6	14 (1)	no	0	1	1	0	no	no	not tested
7	205 (17)	no	> 2	0	0	0	yes	yes	yes
8	195 (16)	no	1	0	0	1	Lost to follow-up	Lost to follow-up	not tested
9	80 (6)	no	1	0	0	0	yes	yes	not tested
10	0 (0)	yes	0	2	0	0	Lost to follow-up	Lost to follow-up	not tested
11	30 (2)	no	0	0	0	1	no	no	not tested
12	199 (16)	no	> 2	0	0	0	no	no	not tested
13	54 (4)	no	> 2	0	0	0	no	no	not tested
14	9 (0)	no	0	1	0	0	no	no	not tested
15	96 (8)	no	> 2	0	0	0	no	no	not tested
16	54 (4)	no	> 2	0	0	0	no	no	not tested
17	122 (10)	no	> 2	0	0	1	yes	yes	not tested
18	73 (6)	yes	0	0	0	1	Lost to follow-up	Lost to follow-up	not tested
19	122 (10)	yes	0	0	0	1	Lost to follow-up	Lost to follow-up	not tested

## Discussion

Our study demonstrates a high frequency of periorbital infections in pediatric patients with PVL + SA associated SSTI. Recurrent hordeola were observed in 18% of children with PVL + SA colonisation. Other manifestations comprised conjunctivitis, preseptal cellulitis and eyelid abscesses. In cases with successful and sustained decolonisation of PVL + SA no further episode of an eye infection occurred.

A recent study of periorbital and orbital cellulitis from the USA (*n* = 85, children) by Foster et al. [24] found PVL + SA evidence in 85% (*n* = 72) cases. In another study (*n* = 49, patients of all ages) by Blomquist et al. [25] the most common eye manifestations of CA-MRSA infection were preseptal cellulitis/lid abscesses and conjunctivitis. Hordeola were not evaluated in this study. A case series (*n* = 9, adult patients) from the USA by Rutar et al. [26] showed the capacity of PVL + MRSA to cause severe eye infections. Nadig et al. [27] detected a high number (*n* = 22 of 33) of PVL + MRSA in a study on eye infections in patients of all ages in India. Two case reports from the UK by Alaghband et al. [22] and from the USA by Rutar et al. [26] presented patients with severe orbital infections resulting in blindness or bacteraemia in otherwise healthy adults. Tsironi et al. [21] reported the case of a previously healthy neonate who developed orbital cellulitis with PVL + MRSA. Sueke et al. [28] reported 9.5% PVL+SA in S. aureus isolates from bacterial keratitis in the United Kingdom.

Our data suggests that not only pediatric patients with severe but also those with recurrent but non-threatening periorbital infections (especially hordeola) should be tested for nasopharyngeal PVL + SA colonisation. In case of PVL + SA detection decolonisation measures should be recommended as described by Gillet et al. [29].

PVL detection depends on accurate sampling, culture and PCR conditions [30]. This should be taken into consideration in patients with typical symptoms but no evidence of PVL + SA.

Also, routine testing for nasopharyngeal or conjunctival colonisation with PVL + SA in patients with a history of SSTI or eye infections may be limited by significant costs of lukS/lukF PCR, especially for primary care physicians with limited budgets.

Therefore, decolonization measures can occasionally be considered in patients with a classic history of recurrent SSTI when testing for PVL + SA is not feasible or negative.

For our study, we evaluated children who presented with SSTI and were than assessed for a history of prior eye infections. Because of this selection bias we do not know the extent of PVL + SA colonisation in children with eye infections but no history of SSTI. Further study of children with recurrent eye infections could determine the frequency of PVL + SA. As a further limitation of our study, we did not include routine screening of conjunctival swabs, lid abscess material or hordeolum tissue for PVL + SA. Further studies with microbiologic testing of conjunctival swabs from symptomatic patients or tissue obtained from hordeola are required to prove the causal relationship between PVL + SA and recurrent eye infections, especially hordeola.

## Conclusion

Our findings suggest a frequent involvement of periorbital skin in children with PVL + SA infections. Pediatric patients with recurrent periorbital infections might benefit from PVL + SA screening and staphylococcal decolonisation procedures.

## Abbreviations

CA: community aquired
EUCAST: European Committee on Antimicrobial Susceptibility Testing
MRSA: methicillin-resistant *Staphylococcus aureus*
MSSA: methicillin-sensitive *Staphylococcus aureus*
PCR: polymerase chain reaction
PVL: Panton-Valentine Leukocidin
SA: *Staphylococcus aureus*
SSTI: skin and soft tissue infections
